# *In vitro* and *in vivo* antiviral activity of monolaurin against *Seneca Valley virus*

**DOI:** 10.3389/fvets.2023.980187

**Published:** 2023-01-27

**Authors:** Bo Su, Yingjie Wang, Shanqiu Jian, Huaqiao Tang, Huidan Deng, Ling Zhu, Xiaonan Zhao, Jian Liu, Huangzuo Cheng, Lina Zhang, Youjun Hu, Zhiwen Xu

**Affiliations:** ^1^College of Veterinary Medicine, Sichaun Agricultural University, Chengdu, China; ^2^Innovation Center of Guangdong Nuacid Biotechnology Co., Ltd., Qingyuan, China; ^3^College of Animal Science and Technology, Jiangxi Agricultural University, Nanchang, China

**Keywords:** MCFA, antiviral agent, monolaurin, SVV, inflammatory response

## Abstract

**Introduction:**

Surveillance of the *Seneca Valley virus* (SVV) shows a disproportionately higher incidence on Chinese pig farms. Currently, there are no vaccines or drugs to treat SVV infection effectively and effective treatment options are urgently needed.

**Methods:**

In this study, we evaluated the antiviral activity of the following medium-chain fatty acids (MCFAs) or triglycerides (MCTs) against SVV: caprylic acid, caprylic monoglyceride, capric monoglyceride, and monolaurin.

**Results:**

*In vitro* experiments showed that monolaurin inhibited viral replication by up to 80%, while *in vivo* studies showed that monolaurin reduced clinical manifestations, viral load, and organ damage in SVV-infected piglets. Monolaurin significantly reduced the release of inflammatory cytokines and promoted the release of interferon-γ, which enhanced the viral clearance activity of this type of MCFA.

**Discussion:**

Therefore, monolaurin is a potentially effective candidate for the treatment of SVV infection in pigs.

## Introduction

*Seneca Valley virus* (SVV) belongs to the genus *Senecavirus* in the family *Picornaviridae*. Phylogenetic analysis of the whole-genome sequence of *Senecavirus* A shows that it is closely related to members of the genus *Cardiovirus* (1). In 2015, Brazilian scientists isolated the complete genome of SVV from vesicular fluid and serum of pigs with vesicular disease and elucidated that SVV infection was associated with idiopathic vesicular disease in pigs (2). Subsequently, many other countries have also reported cases of pigs infected with SVV, where newborn piglets are more vulnerable to SVV infection. The main clinical manifestations of SVV infection in pigs are blisters and ulcers on the hoofs and snout (3, 4). Clinical symptoms can be similar to foot-and-mouth disease, swine vesicular disease, and vesicular stomatitis, with a potential impact on the immune system of pigs (5). The virus is shed through the oral cavity, nasal secretions, and feces with a viral shedding duration of ~28 days after infection (3). Presently, SVV is sporadically and locally prevalent, but its transmission mechanism is not completely clear. Currently, there is no vaccine or specific drug available for the prevention and treatment of SVV infection in pigs. Therefore, the control of SVV in pigs depends on the hygiene measures implemented on pig farms.

Replacing antibiotics in animal feed with biologically active substances has become a hot topic in China. Medium-chain fatty acids (MCFAs) are a class of saturated fatty acids containing 6–12 carbon atoms. Even-numbered carbon MCFAs, such as caproic acid (C6), caprylic acid (C8), capric acid (C10), and lauric acid (C12), are found in natural foods, such as coconut oil, palm kernel oil, and milk. MCFAs undergo esterification with glycerol to form triglycerides, known as medium-chain fatty acid triglycerides (MCTs). In addition to being a source of energy, MCFAs can also improve intestinal morphological structure and growth, prevent infection, regulate immunity, and act as an alternative to antibiotics (6, 7). Both MCFAs and MCTs exhibit strong bacteriostatic activity against a variety of pathogens, including gram-positive and gram-negative bacteria, viruses, fungi, algae, and protozoa (8, 9).

The antimicrobial properties of fatty acids have been reported extensively in the literature (10). Previously, studies have confirmed the antiviral activity of free MCFAs such as capric, lauric, myristic, and long-chain unsaturated oleic, linoleic and linolenic acids against vesicular stomatitis virus (VSV), herpes simplex virus (HSV) and visna virus (11). Other studies reported similar antiviral activity of MCFAs, together with their alcohol and monoglyceride derivatives, against HSV 1 and 2 (12). Research also showed that MCFAs (caprylic, capric, and lauric acids) and monolaurin can inhibit ASFV in liquid conditions and reduce Asfivirus (ASFV) infectivity, which may help to prevent disease progression and virus transmission (13). On the one hand, SVV is a small, non-enveloped picornavirus, unknown until 2002 when it was discovered incidentally as a cell culture contaminant, and the family Picornaviridae also contains foot-and-mouth disease virus (FMDV) and swine vesicular disease virus (SVDV). On the other hand, since the vesicular lesions caused by SVV infection are clinically similar from those caused by other vesicular disease viruses, such as FMDV, SVDV, VSV and vesicular exanthema of swine virus (VESV). Thus, we evaluated the antiviral activity of MCFAs or MCTs against SVV. In this study, the anti-SVV activity of selected MCFAs and MCTs was evaluated *in vitro*, and the most effective compound was selected and tested *in vivo*. The clinical symptoms, viral load, and proinflammatory cytokines were recorded and analyzed to evaluate the anti-SVV activity of monolaurin. Our results provide a reliable basis for the potential clinical use of monolaurin for the treatment of SVV infection in pigs.

## Materials and methods

### Samples and reagents

BHK-21 cells and the Seneca virus A strain SVV-SC-MS (complete genome GenBank: MN700930.1) were obtained from the Animal Biotechnology Center (ABTC) at Sichuan Agricultural University School of Veterinary Medicine. Fetal bovine serum, cell culture medium (DMEM), trypsin, and PBS buffer were purchased from Solarbio (Beijing Solarbio Science and Technology Co., Ltd., Beijing, China); DMSO was purchased from Sigma (USA); the CCK8 kit (code: Beyotime. C0038) used in this study was purchased from Beyotime Biotechnology Co., Ltd. Caprylic, caprylic monoglyceride, capric monoglyceride, and monolaurin were prepared by Guangdong Nuacid Biotechnology Co., Ltd. The PrimeScript™ RT reagent Kit (Perfect Real Time), DNA/RNA extraction kit, and TB Green^®^ Premix Ex Taq™ (Tli RNaseH Plus) were purchased from Takara (Dalian) Engineering Co., Ltd.

### Maximum nontoxic dose

BHK-21 cells were cultured in a 96-well plate at 37°C under 5% CO_2_ for 24–36 h until the cells grew into a monolayer. Caprylic, caprylic monoglyceride, capric monoglyceride, and monolaurin were dissolved in DMSO independently to prepare a 10 mg/mL stock solution. A cell maintenance solution of 100 μg/mL was prepared from the stock solution for these four test MCFA, whereafter a total of 11 concentration gradients were prepared from the cell maintenance solution following 2-fold gradient dilution. The concentrations of the cell maintenance solution for these four test MCFA were 100, 50, 25, 12.5, 6.25, 3.125, 1.563, 0.781, 0.391, 0.195, 0.098, and 0.049 μg/mL. Supernatant from all wells of a 96-well-plate with monolayer BHK-21 cells was discarded, and the MCFA sample solution (100 μL/well) was added. Two percent DMEM and one precebt DMSO controls were also set at the same time. The cells were cultured in an incubator for 48 h at 37°C and 5% CO_2_. A cytotoxicity assay was performed according to the instructions of the CCK8 kit, and the cell viability was calculated. The concentration corresponding to a cell viability >90% was recorded as the maximum non-toxic dose (MNTD), which was used as the working dose for subsequent experiments.

### *In vitro* calculation of viral inhibition rate

A mixture of 1 MNTD and 100 TCID_50_ viral suspension was prepared by mixing the virus solution with the MCFA solution. BHK-21 cells were seeded in a 96-well-plate and grown to monolayers at 37°C in a 5% CO_2_ incubator. The supernatants were discarded, and an equal volume of the viral suspension was added to the wells (100 μL/well) of the experimental group. The 2% DMEM (A) and 1% DMSO (B) controls were also set. The plates were incubated for 1 h at 37°C with 5% CO_2_ in a cell incubator. After incubation, the supernatant was discarded, and 100 μL of the sample solution with a concentration of 1 MNTD or 100 μL of the maintenance solution was added to the corresponding wells and then incubated at 37°C in a 5% CO_2_ incubator. Cell infection was stopped when complete CPE was developed in virus-only control wells (for ~36–48 h post-infection). The supernatants were collected and measured with the CCK8 method, and the virus inhibition rate was calculated.

### *In vivo* evaluation of anti-SVV activity

A total of 25 weaned piglets at 21 days old were obtained from a pig farm (Sichuan gistar group) in Sichuan Province, China. All the piglets were SVV negative for the antigen and antibody test by PCR or ELISA kits (detection methods were established by ATBC). Before the experiment, the animal lab was sterilized with formaldehyde and pasteurizer. All the pigs were cared for according to original farm procedures to prevent stress and bacterial infection in the pigs. The piglets were fed common complete feed. Piglets were first observed for 3 days and then subjected to viral challenge and drug administration. The 25 weaned piglets were divided into 5 groups (*n* = 5), as shown in Table 1.

**Table 1:** Piglet grouping, viral challenge, and drug administration information.

**Group**	***n***	**SVV**	**Monolaurin**	**Treatment** **time**
Control	5	Cell supernatants	Saline	Days 1, 2, and 3
Model	5	10^5^TCID_50_/mL	Saline	
Low	5	10^5^TCID_50_/mL	0.5 g	
Medium	5	10^5^TCID_50_/mL	1 g	
High	5	10^5^TCID_50_/mL	2 g	

### Clinical symptoms

The clinical symptoms of piglets in each group were observed daily, and scores were assigned as follows: fever at 2 points; lethargy at 2 points; decreased feed intake at 1 point; anorexia at 2 points; blisters or ulcers at 2 points; and death as 5 points. Additionally, for every piglet in each group, morning feces and 0.5 mL jugular blood were collected daily for the determination of SVV load. Whenever blisters and crusts appeared or the piglets were on the verge of death, they were immediately sacrificed and necropsied. On day 14, all the remaining piglets were sacrificed, and the lungs, spleens, kidneys, and livers were aseptically collected and fixed in 4% paraformaldehyde.

### Quantitative detection of SVV load in stool and blood by RT–qPCR

The feces and blood samples were thoroughly mixed with 3.0 mL of PBS and then centrifuged at 12,000 r/min for 3 min. Supernatants were collected for RNA extraction. Extracted RNA was reverse transcribed to obtain cDNA, which was added to the PCR master mix as detailed in Table 2, and loaded into a fluorescent quantitative PCR machine to detect the SVV viral load under the following thermocycling conditions: 40 cycles of denaturation at 95°C for 30 s, annealing at 95°C for 5 s, and elongation at 58°C for 30 s. At the end of the amplification, melting curve analysis was performed from 65 to 95°C with 0.5°C per second.

**Table 2:** The PCR master mix used for quantitative SVV detection from piglet stool and blood.

Primer F: AGGTACTGGAGAAGGACGCT	0.5 μL
Primer R: GGTTGACGTACAGGCCGAAA	0.5 μL
ddH2O	3 μL
SYBR Green Premix Ex TaqII	5 μL
cDNA	1 μL
Total	10 μL

### Histopathology

Briefly, the Lung, spleen, liver, and kidney tissues were fixed in 4% paraformaldehyde for 36 h and then embedded in paraffin. According to standard method, tissue sections (4 μm), were stained with Hematoxylin and Eosin (H&E) for histopathological examination. Finally, histological lesions were recorded with a light microscope (OLYMPUS, Japan) at 400× magnifications.

### *In vivo* detection of inflammatory cytokines

Piglet venous blood was collected at 0 and 3 dpi. The expression of IL-6, IL-8, IL-10, IL-1β, IFN-γ, and TNF-α in the supernatant was detected using ELISA kits according to the manufacturer's instructions (Multisciences (Lianke) Biotech Co., Ltd., Hangzhou, China). The absorbance was measured using a microplate reader at 450 nm. In brief, the samples were added to the wells, followed by the antigen in the samples bounding to the capture antibody, the microplate was washed, the detection antibody was added, and the microplate was washed again. After the substrate was added, the microplate reader detected the colored reaction products and calculated light density (OD) values, which were used to calculate and analyze the amount of antigen in each sample.

### Statistical analysis

Statistical results were expressed as means and standard deviation (SD). Significant differences were determined with one-way analysis of variance (ANOVA), followed by Duncan's multiple range test in SPSS 20.0 (IBM Corp., Armonk, NY, USA). Significance was set at *P* < 0.05.

## Results

### Maximum non-toxic concentration determination

**Figure 1:**
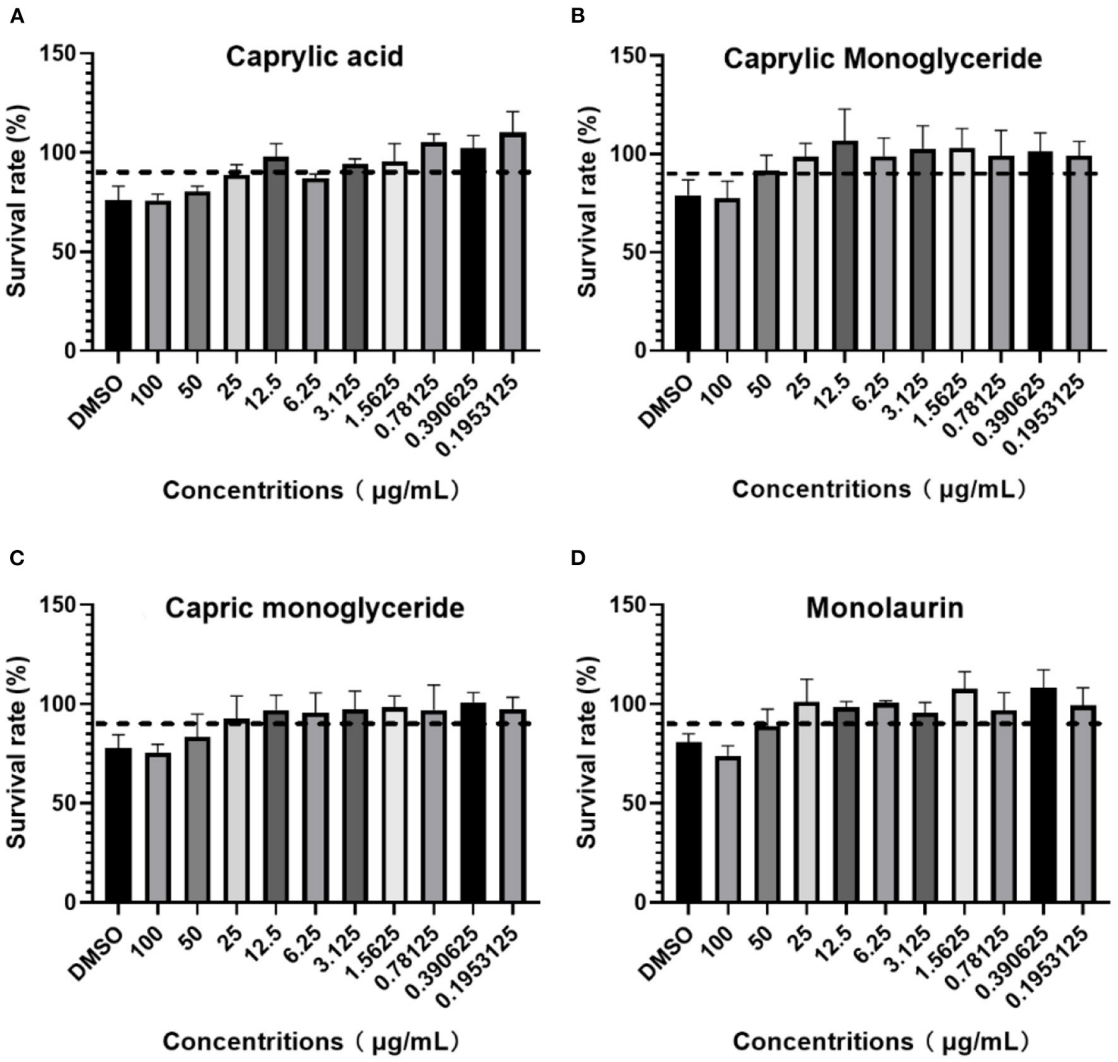
BHK-21 cell survival rate in: **(A)** Caprylic acid; **(B)** Caprylic monoglyceride; **(C)** Carpric monoglyceride; **(D)** Monolaurin.

After adding different concentrations of drugs and culturing for 96 h, the OD_450_ value or cell viability was determined using a CCK-8 kit following the manufacturer's instructions. If the cell viability was >90%, the dose was recorded as MBTD. BHK-21 cells showed good tolerance to glycerol caprylate and monoglycery laurate with an MNTD of 50 μg/mL. Caprylic and capric monoglycerides showed little toxicity to these cells with an MNTD of 25 μg/mL (Figure 1).

### Monolaurin inhibits virus proliferation

**Figure 2:**
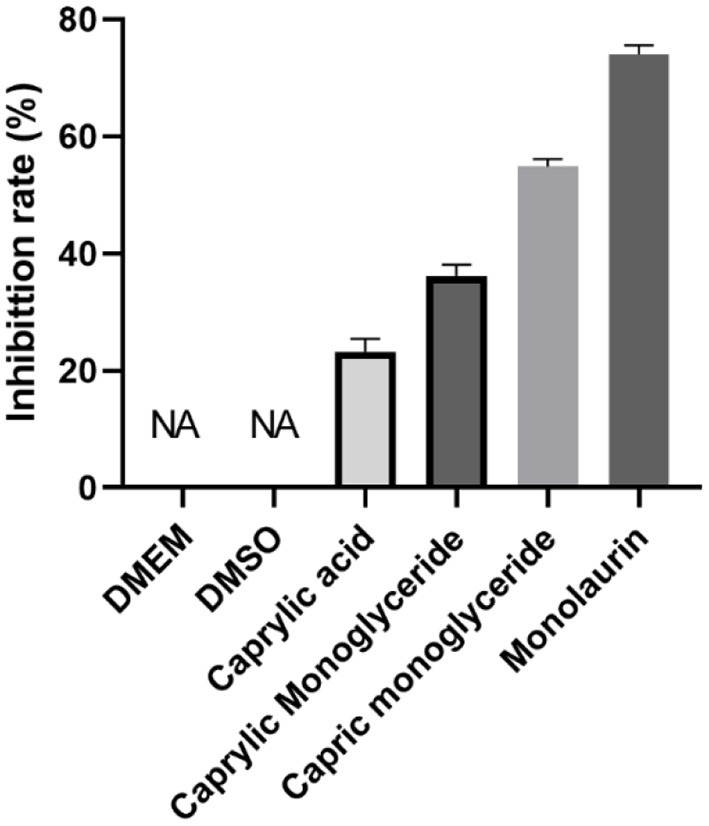
*In vitro* evaluation of the anti-SVV activity of MCFAs.

Although all four treatments had an inhibitory effect on SVV, the inhibitory rate of monolaurin was the highest, with values up to 80% (Figure 2). The anti-SVV activity of caprylic monoglycerides was better than that of capric monoglycerides. Monolaurin was selected for the subsequent *in vivo* tests.

### Clinical symptoms and scores

**Figure 3:**
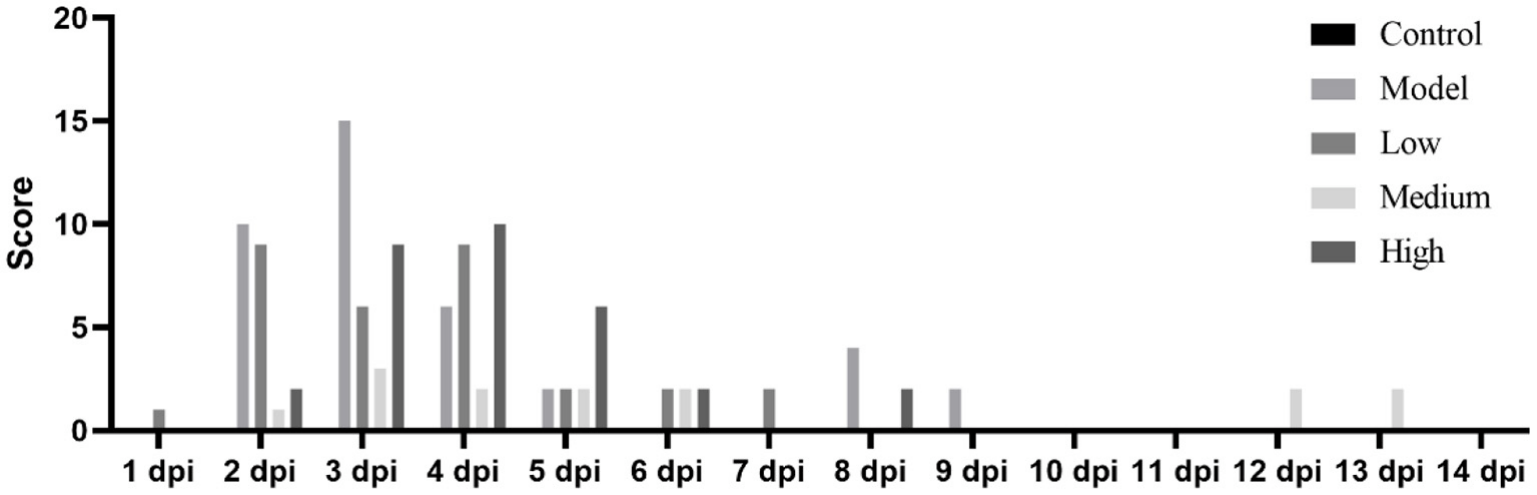
Clinical symptom scores for piglets infected with SVV.

After the piglets were challenged with the virus and treated with monolaurin, the development and progression of their clinical symptoms were observed and monitored continuously for 14 days (Figure 3). One piglet from the low-dose group died at 2 dpi. There were no other piglet deaths recorded in this group on the subsequent days. One piglet from the model group died at 3 dpi. No piglets died in the middle-dose, high-dose, or control groups. The piglets in the low-dose and model groups showed decreased feed intake and symptoms such as anorexia, lethargy, and fever after the virus challenge. These piglets had blisters and ulcers on their snouts and hooves at 7 dpi. Except that erosions, ulcerations, and vesicular lesions of the snout, oral mucosa, and distal limbs, especially around the coronary band, as well as more general symptoms of illness such as fever, lethargy, and anorexia, may be observed from the model group. Hoof sloughing and lameness can also occur. By contrast the clinical symptoms, mental status, and feed intake of the monolaurin-treated piglets were better than those of piglets in the model group. The piglets in the middle-dose and high-dose groups had similar clinical symptoms, with a lower post-challenge score than that of the model group.

### Post-challenge effects of monolaurin on SVV load

**Figure 4:**
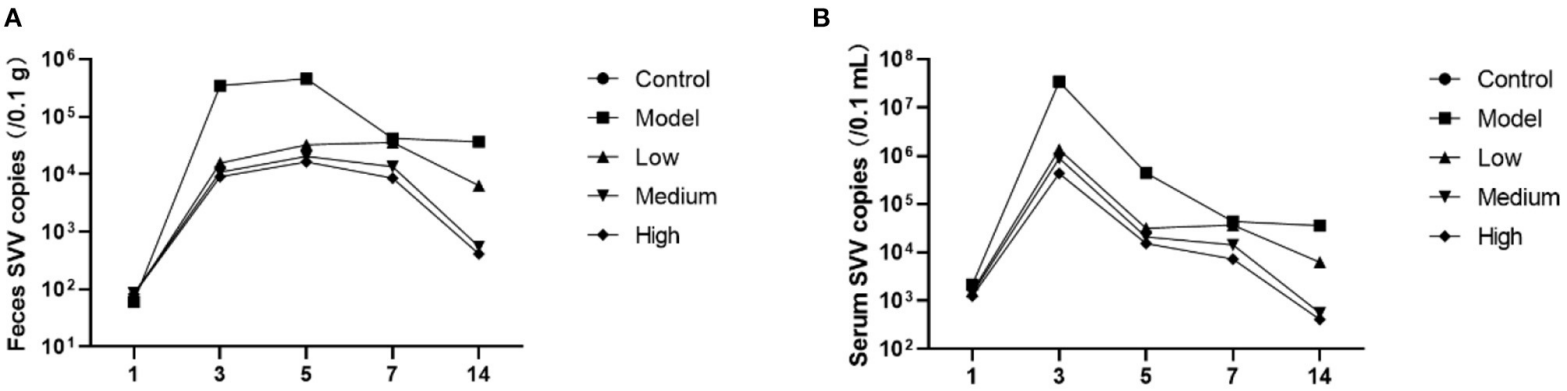
Viral load in the feces and blood of piglets infected with SVV. **(A)** Viral load in feces; **(B)** Viral load in blood.

Piglet viral load peaked at 3 dpi and then continued to decline (Figure 3). Monolaurin reduced the viral load in the feces and blood of piglets infected with SVV in a dose-dependent manner (Figure 4). The treatment with high-dose Monolaurin was the most effective for viral load in fecal and blood of SVV-infected piglets. Compared with fecal viral load of SVV infected piglets, blood viral load decreased more significantly after 3 dpi (Figure 4B).

### Histopathological examination

**Figure 5:**
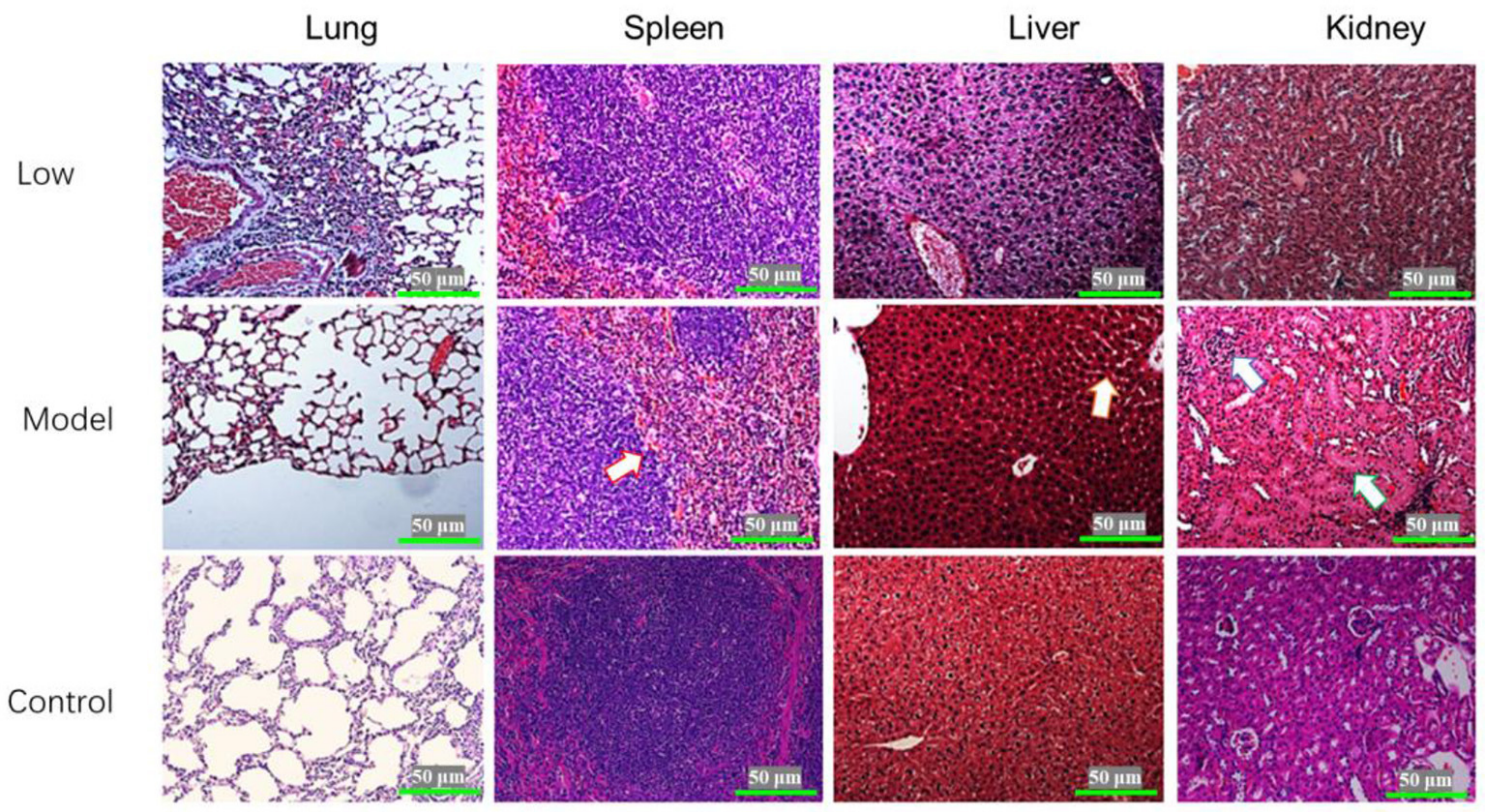
Histopathological examination of the lungs, spleen, liver, and kidneys of piglets infected with SVV (HE×400). The arrow on Model-spleen indicated the hemorrhage diffuse; the arrow on Model-liver indicated the cell swelling; and the arrows on Model-kidney indicated the glomerular atrophy and tubular swelling.

Blisters and ulcers manifested on the snout and hoofs at 7 dpi. Pigs in the model and low-dose groups exhibited the following clinical manifestations: parts of the lung were atrophied, the alveolar septum was thickened, and the rest of the lung tissues had compensatory emphysema. In the model group, spleens showed diffuse hemorrhage, severe swelling of hepatocytes, partial cell necrosis, glomerular atrophy, and partial shedding of the renal tubular epithelium. There were no significant changes in the spleens, livers, and kidneys of pigs in the low-dose group. The pigs in the middle-dose and high-dose groups had alveoli without obvious lesions, while their spleens, kidneys, and livers had clear structures but no obvious lesions (Figure 5).

### *In vivo* detection of inflammatory cytokines

**Figure 6:**
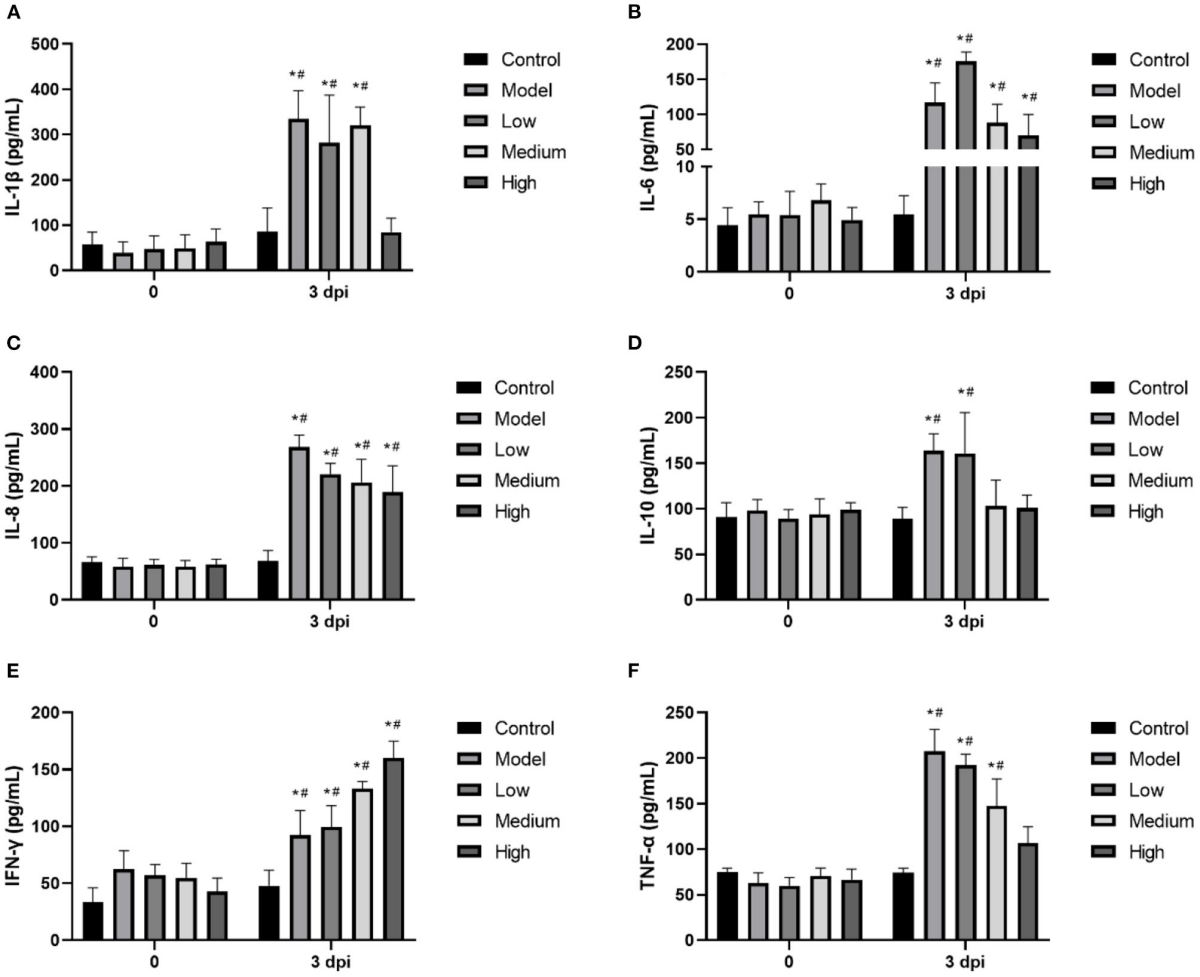
**(A–F)** The relative mRNA expressions of cellular inflammatory factors IL-1β, IL-6, IL-8, IL-10, IFN-γ. and TNF-α in piglet blood. Effects of monolaurin on cytokine release in piglet blood infected with SVV: *p < 0.05 *vs*. 0 dpi, ^#^*p* < 0.05 *vs*. control.

Proinflammatory cytokines were significantly increased by SVV infection, and treatment with monolaurin showed some degree of anti-inflammatory activity (Figure 6). High doses of monolaurin significantly decreased the levels of IL-1β, IL-10, and TNF-α to levels (*p* < 0.05), without a significant difference compared with the corresponding control groups (*p* > 0.05). However, with the increase of the monolaurin dose, the trend of the increase of IFN-γ level is more obvious. So, high doses of monolaurin significantly increased the amount of IFN-γ in a dose-dependent manner.

## Discussion

Monolaurin, a monoglyceride formed from 12-carbon saturated fatty acids and glycerol, is naturally found in coconut oil, palm oil, and breast milk, and is a safe and highly effective monoglyceride with bacteriostatic activity (14). Monolaurin has broad antibacterial activities, including inhibiting bacterial growth, reducing the production of exotoxin, and forming biofilms (15). As a lipid, monolaurin can bind to the phospholipid bilayer of bacteria and disrupt the normal physiological processes of the bacteria, thereby inducing a bacteriostatic effect. Monolaurin is also reported to have a strong inhibitory effect on the growth and reproduction of gram-positive bacteria such as *Staphylococcus aureus, Listeria monocytogenes, Helicobacter pylori, Bacillus*, and *Campylobacter jejuni*, among others (16). Furthermore, monolaurin can block the release of gram-positive bacterial exotoxins (such as enterotoxins and streptococcal pyrogenic exotoxins, etc.) (17). Monolaurin can also bind to the lipid bilayer membrane enveloped viruses and inhibit viral activity by compromising viral integrity and infectivity. Monolaurin has shown a good inhibitory effect on some enveloped viruses, such as the HSV, influenza virus, PRRS virus, and porcine epidemic diarrhea virus (18, 19). In this study, we show that monolaurin has a strong inhibitory effect on SVV even though it is a non-enveloped virus. However, the anti-SVV mechanism of monolaurin needs further research.

Even though SVV causes blisters and ulcers on the snout and hoofs of pigs, there are only a few reports on autopsy symptoms and microscopic pathogenesis of this viral infection. Pathological experiments in this study showed lesions in the lungs, livers, spleens, and kidneys of infected piglets. Preventing SVV from destroying the integrity of the intestinal barrier helps to reduce the damage of the virus. Monolaurin has great potential for application in animal health, as it promotes growth and gut health. For example, some studies have found that monolaurin can significantly improve the growth performance of weaned piglets (20, 21). Additionally, dose-related monolaurin has been found to improve body weight, regulation of gut microbiota, and systemic inflammation in mice fed a low-fat diet (22). These studies show a significant positive correlation between monolaurin and the increased abundance of probiotics, such as *Lactobacillus reuteri* and *Ruminococcus gnavus* (22). Normal intestinal flora is necessary for the integrity of the tight junctions of the intestinal tract. Here, monolaurin significantly improves the health of the intestinal tract, which reduces the chance of viruses invading the intestinal epithelial cells and the bloodstream. Our findings corroborate the findings of these previous studies, as we found that monolaurin-treated pigs had significantly reduced viral loads in their blood and feces as well as reduced clinical symptoms associated with SVV infection. During the trial, one piglet from the low-dose group died at 2 dpi, one piglet from the model group died at 3 dpi. No piglets died in the middle-dose, high-dose.

Many viruses can induce inflammatory responses and even cause an inflammatory factor storm (23). The mechanism is the excessive activation of immune cells by increasing intracellular inflammatory factors, including interleukin, TNF-α, and complement protein molecules (24). The storm-like suicide attack induced by pathogenic microorganisms in infected cells can cause bystander damage to other tissues by increasing vascular permeability and circulatory disorders, which can even result in multiple organ functional failure (MOF) (25). Usually, inflammation is a protective immune response that is conducive to clearing pathogenic microorganisms. However, uncontrollable excessive inflammation can cause autoimmune damage (26). In this study, we observed that SVV infection induced the release of many inflammatory cytokines, including IL-1β, IL-6, IL-8, IL-10, and TNF-α, triggering an inflammatory cytokine storm. Previous studies have found that monolaurin affects the lipid dynamics of human T cells and regulates T-cell signaling and the release of functional factors. It also inhibits the immune response that is overactivated by the virus, thereby reducing the amount of SVV-induced inflammatory cells (27). *Seneca Valley* virus infection can reduce the level of IFN-γ in the serum, hence reducing the antiviral activity of the body. On the other hand, monolaurin can increase the level of IFN-γ, possibly explaining one of the anti-viral mechanisms of MCFAs.

The results from this study support the efficacy of Monolaurin against SVV. Data suggest that monolaurin block virus proliferation and systemic inflammation response. Despite the promising results, our sample sizes were small and it is not clear whether other mechanisms are involved in the regulation of the efficacy of antiviral therapies. A large-scale evaluation of the anti-viral potential and mechanism of monolaurin should be the subject of future studies.

## Data availability statement

The original contributions presented in the study are included in the article/supplementary material, further inquiries can be directed to the corresponding authors.

## Ethics statement

The animal study was reviewed and approved by the Animal Ethical Committee of Sichuan Agricultural University.

## Author contributions

BS, YW, and SJ conceived of the presented idea. HT and HD developed the theory and performed the computations. All authors contributed to the article and approved the submitted version.
